# Burden of migraine among university students in the Middle East and North Africa: a cross-sectional study of prevalence, mental health comorbidities, and disability

**DOI:** 10.1186/s12889-025-24293-9

**Published:** 2025-10-27

**Authors:** Ahmed Amir Samir, Ahmed Samy Elgammal, Abdelrhman Mahmoud Alieldeen, Eman Gomaa Allam, Imen ketata, Emna Ellouz, Rawan Gameel Hekal, Hossam Salameh, Nasrouha Didda, Naima Kebir, Abderrhman Abdelfattah Selim, Hussein Zeyad Atshan, Aiman Awad Bashir, Riad Azzam Kouzeiha, Hanadi Alayiab, Ibrahim Ali Kabbash

**Affiliations:** 1 Faculty of Medicine, Al-Azhar University, Nasr city, Cairo, Egypt; 2 Faculty of Medicine, Tanta University, Tanta, Egypt; 3 Faculty of Medicine, Delta University for Science and Technology, Dakahlia, Egypt; 4 Faculty of Medicine for Girls, Al-Azhar University, Nasr City, Cario Egypt; 5 Neurology Department of Gabes, Faculty of Medicine, University Hospital of Gabes, University of Sfax, Sfax, Tunisia; 6 Faculty of Medicine, Menoufia University, Menoufia, Egypt; 7 Faculty of Medicine and Health Sciences, An-Najah National University, Nablus, Palestine; 8 Department of Clinical Laboratory Diagnostics, Faculty of Medicine, Astrakhan University, Astrakhan, Russia; 9 Faculty of Medicine, Oran1 University, Oran, Algeria; 10 Faculty of Medicine, Alexandria University, Alexandria, Egypt; 11 Faculty of Medicine, University of Baghdad, Baghdad, Iraq; 12 Faculty of Medicine, University of Khartoum, Khartoum, Sudan; 13 Faculty of Medical Sciences, Lebanese University, Hadath Campus, Beirut, Lebanon; 14 Faculty of Human Medicine, Benghazi University, Benghazi, Libya; 15 Professor of Public Health & Community Medicine, Faculty of Medicine, Tanta University, Tanta, Egypt

**Keywords:** Migraine, Prevalence, Mental health, University students, Academic life, MENA region

## Abstract

**Background:**

Migraine is a prevalent and debilitating neurological condition that significantly impact the academic lives of university students. Despite its high prevalence, migraine is often underreported, underdiagnosed, and inadequately managed particularly in the Middle East and North Africa (MENA) region.

**Objective:**

This study aimed to determine the prevalence of migraine identified by screening, related disability, and psychological comorbidities among university students in the MENA region.

**Methods:**

We conducted a multinational cross-sectional study among university students in 11 low and middle-income countries in the MENA region, using an anonymous self-administered questionnaire. The convenience and snowball sampling methods were used. This study utilized a validated questionnaire to collect data on migraine frequency, characteristics, disability, associated depression, anxiety, and triggers.

**Results:**

The present study screened a total of 5,954 students for migraine. Of them, 26.1% screened positive for migraine. Iraq had the highest prevalence 38.9%, followed by Algeria 31.5%, and the lowest was in Egypt 19.9% and Morocco 18.4%. Common migraine triggers included sleeping disturbances 59.7%, noise 47.4%, and sun exposure 45.6%. Among positive cases, 23.2% had severe disability, 29.9% had moderate anxiety, and 72.5% had severe depression. The main predictors of migraine were females, older age, and non-medical field university students. Adequate hydration, sufficient sleep, regular physical activity, higher fluid intake, and extended study hours were associated with a lower risk of migraine. Daily caffeine consumption was associated with increased migraine risk. A modest negative relationship was found between academic success and migraine disability score.

**Conclusion:**

Our results demonstrated a high proportion of students screened positive for migraine with significant associations to depression, anxiety, and disability. These findings highlight the need for targeted interventions to increase awareness about migraine-related comorbidities, screening programs to help in early detection, and lifestyle modification. Universities should develop and implement coping strategies to support affected students.

**Supplementary Information:**

The online version contains supplementary material available at 10.1186/s12889-025-24293-9.

## Introduction

Migraine is a widespread debilitating neurological condition, characterized by episodic headache attacks, that usually last 4–72 hours, and typically manifest as unilateral throbbing, pulsing head pain accompanied by symptoms such as nausea, vomiting, fatigue, and hypersensitivity to light, sound, and smells [1, 2]. Stress, certain foods, weather, dehydration, caffeine, changes in sleep patterns, smoking, medication usage, and abrupt exposure to strong odors or light are common triggers for these episodes [3]. Migraine is influenced by genetic, regional, cultural, and environmental factors [4]. Migraine represents a major health problem that remains under-recognized and under-treated, and its global prevalence has significantly increased in recent decades, currently affecting more than 15% of the general population [5].

According to The Global Burden of Disease (GBD) study 2019, migraine is the second most prevalent condition that causes impairment in young males and first in young females, and it is the leading cause of the years lived with disability (YLDs) among young women aged 15-49 years [6]. Migraine typically begins around puberty and is three times more prevalent in females. This could be due to genetic predisposition, hormonal differences between sexes, and varying responses to stress and pain [7, 8]. Migraine primarily impacts people between the ages of 25 and 55, which are the most productive working years [9].

Migraine affects a significant proportion of the Middle Eastern population. A previous systematic review of 23 prior studies assessing the burden of migraine among the general population from Arab countries between 1990 and 2019 showed a wide variation in migraine prevalence, ranging between 2.6% and 32%. Additionally, female predominantly and clinical characteristics correspond to those in global statistics [10]. Similarly, another review revealed the same prevalence among Middle Eastern countries with the average age ranging from 27 to 37.5 years among patients with migraine, and females exhibiting a 2- to 2.5-fold higher prevalence. Additionally, depression, anxiety, and irritable bowel syndrome were the most common comorbidities [11].

Migraine places a burden on patients who suffer from it, including significant personal misery, disability, impact on family life, reduced quality of life, functional impairment, and increased financial cost. Headache-related productivity impairment can impact individuals'employment and/or assurance, financial condition, relationships, and psychological health [1, 12]. Various comorbidities linked to the occurrence of migraines such as asthma, fibromyalgia, allergy, obesity, sleep disorders, gastrointestinal disorders, hypertension, and poor dietary habits [13]. On top of that, migraine is associated with a wide variety of psychiatric disorders and the bidirectional relationship between psychiatric problems and migraine is subject to increasing research and attention [14]. Anxiety and depression are the most commonly associated comorbidities among migraine sufferers and were significantly independently associated with the increased risk of migraine and migraine-related burden [15].

University students face psychological and physical stressors, such as demanding coursework, exams, academic, and performance pressure, which all trigger migraines and negatively influence educational performance, productivity, and quality of life [16]. Migraine has been correlated with absenteeism, impaired daily activities, missed school days, and poor academic performance [17]. A recent meta-analysis showed that the prevalence of migraine among university students was 19% [95% CI: 16%–22%]. This prevalence was 16% [95% CI: 13%–20%] in Asia, and 15% [95% CI: 10%–23%] in Africa [18].

Despite the studies showing that migraine has grown considerably, less is known about the prevalence of migraine in is in the Middle East and North Africa (MENA) region, especially among university students in low- and middle-income countries. Also, there is a scarcity of comprehensive data on the epidemiology of the burden of this condition, while existing literature frequently focuses on specific countries. Considering this gap, we aim to determine the prevalence of migraine using the quick Migraine Screening Questionnaire (MS-Q) among university students in MENA low-and middle-income countries and examine their characteristics, associated triggers, disability, and mental health comorbidities. This study's findings will enhance our understanding of migraine epidemiology in the region and guide the creation of targeted interventions aimed at preventing and managing this debilitating condition among university students.

## Materials and methods

### Study design

We conducted a multinational cross-sectional study between August and December 2024, among university students in 11 low- and middle-income countries in the MENA region, including (Egypt, Jordan, Palestine, Syria, Iraq, Libya, Algeria, Morocco, Sudan, Yemen, and Lebanon). We collected data by using an online self-administered questionnaire. The present study complied with the Strengthening the Reporting of Observational Studies in Epidemiology (STROBE) checklist [19].

### Eligibility criteria

Undergraduate university students, of both sexes, from all academic years and various faculties, and who were able to complete the survey in Arabic language were invited for participation in this study. Participants who couldn’t give their informed consent, and students with a history of secondary headaches such as head trauma, sinusitis, neurological conditions, or systemic illness that causes headaches were excluded.

### Sampling and sample size calculation

To recruit the study participants, convenience and snowball sampling methods were utilized. We calculated the sample size using the trademark of the Center for Disease Control and Prevention (CDC) Epi Info statistical software 7.2.6. version, with an expected frequency of 19% based on the estimated global prevalence of migraine among university students [18], and a confidence interval of 95%, a 4% acceptable margin of error. The minimum sample size was 369 students for each country.

### Study tools

We designed a structured questionnaire adopted from existing Arabic validated tools. The questionnaire consists of five sections: The participants’ sociodemographic and lifestyle characteristics addressed in the first section, including age, gender, marital status, monthly family income, field of study, last academic grade, sleeping, physical activity and studying hours, smoking cigarettes, family history of migraine, and chronic illnesses. The second section utilized the MS-Q [20]. MS-Q is a quick, self-administered questionnaire and consists of five questions regarding the frequency and characteristics of headaches along with the absence or presence of symptoms of migraine. A score of zero was given for every negative answer, and one for each positive answer. A cut-off point of ≥ 4 was established, indicating suspicion of migraine. For the Arabic-speaking population, the scale was translated and validated previously by experts and showed good reliability and internal consistency with the Cronbach α coefficient of 0.81 [20]. The third section comprised the Migraine Disability Assessment Score (MIDAS) to assess the degree of disability [21]. Many studies used MIDAS as a tool to assess headache-related disability for over 3 months. The MIDAS included five questions and evaluated impairment in school/work, housework, and social activities, by determining the number of missed days in these dimensions with the total score reflecting overall disability. We categorized the score as follows: Grade I (0–5) has minimal or no impairment, grade II (6–10) has a mild disability, grade III (11–20) has a moderate disability, and grade IV (21 or more) has severe disability. The Arabic version of the MIDAS was developed in a previous study with Cronbach’s Alpha = 0.812 [21]. The current study utilized the Generalized Anxiety Disorder 7-item (GAD-7) scale in the fourth section to measure anxiety levels [22]. GAD-7 includes 7 items scored from 0 (not at all) to 3 (almost every day). Scores range between zero and 21, with higher scores suggesting more severe anxiety symptoms. The total anxiety score is divided into minimal anxiety (0–4), mild (5-9), moderate (10-14), and severe anxiety (15-21) [23]. Finally, the fifth section includes the Patient Health Questionnaire (PHQ-9) to assess depressive symptoms [22]. This 9-item scale measures the frequency of depression symptoms over the past two weeks on a scale from zero (not at all) to three (nearly every day). The scores range from 0 to 27. The total depression score is subdivided into minimal or none (0–4), mild (5-9), moderate (10-14), moderately severe (15-19), and severe (20-27) [24]. Both the Arabic versions of PHQ-9 and GAD-7 were translated and validated previously among university students with Cronbach’s Alpha of 0.857, and 0.763, respectively [22].

### Pilot study

Before the beginning of data collection, a panel of experts reviewed the full version of the questionnaire for comprehensiveness and relevance. To further refine the survey, we conducted a pilot study among 140 university students (54 males and 86 females) representing nine selected target countries and covering various academic years and fields. The sample size used was sufficient to evaluate cultural and linguistic background independence for clarity, comprehension, and reliability. Their results were not included in the study results. Feedback from the collaborators and respondents on the questions’ clarity, understanding, and language were collected by open-ended questions, and writing comments, and reviewed before data collection. In addition, the reliability and internal consistency of the MS-Q and MDAS were tested to help validate the tool’s acceptability and usability in the MENA student population, and we calculated Cronbach’s alpha, which was 0.72 and 0.71 respectively.

### Data collection and handling

We collected data by utilizing an online link to the web-based questionnaire that was developed using the “Google Forms” platform. To ensure the quality of the data collection process, we assigned a representative group of collaborators from each selected country to collect the data from their national society. Before the data collection process, we trained them on survey distribution strategies and data collection protocols. With the assistance of Migraine collaborative group, the survey was distributed to students through various online platforms accessible to university students including university social media channels, websites, student portals, online student forums, and discussion groups. At the beginning of the survey sheet, we provided a detailed description of the goals and objectives of the study with informed consent with choices to advance or quit the questionnaire. We activated a restriction option for a single response in the Google form setting to prevent duplicate responses via email.

### Ethical considerations

This study followed the principles of the Declaration of Helsinki (1964, last revised in 2013) in its entirety [25]. Participation in this study was voluntary, and participants provided their informed consent electronically before completing the survey, after a thorough description of the study that emphasized their anonymity and confidentiality. Ethical approval was obtained from the institutional review board committee (IRB) at the Faculty of Medicine, Tanta University, (Approval number 36264PR848/9/24). The Approval obtained from Tanta University was reviewed and accepted by other universities.

### Statistical analysis

The statistical analyses were conducted using R version 4.3.1 (2023-06-16 ucrt). Categorical variables were summarized as frequency and percentage. Modified Poisson regression models were used to estimate unadjusted and adjusted prevalence ratios (PRs) and 95% confidence intervals (CIs) for the binary outcome of migraine. Independent variables were included in the multivariable model based on statistical significance (*p*<0.05), clinical relevance, and existing literature. We conducted univariable analysis for demographics, lifestyle factors, and comorbidities. Chi-square tests were used to compare sociodemographic, lifestyle, and psychological distress among MENA countries groups by socioeconomic level. Spearman correlation was used to examine the relationship between migraine disability and academic performance. PRs with 95% CIs were reported for each predictor. Statistical significance was set at *p* < 0.05 for all analyses. Additionally, we performed a sensitivity analysis using logistics regression models to assess the robustness of associations and prevalence estimates. The univariate and multivariate logistics regression models are depicted in the supplement appendix Table S1-4.

## Results

**Fig. 1:**
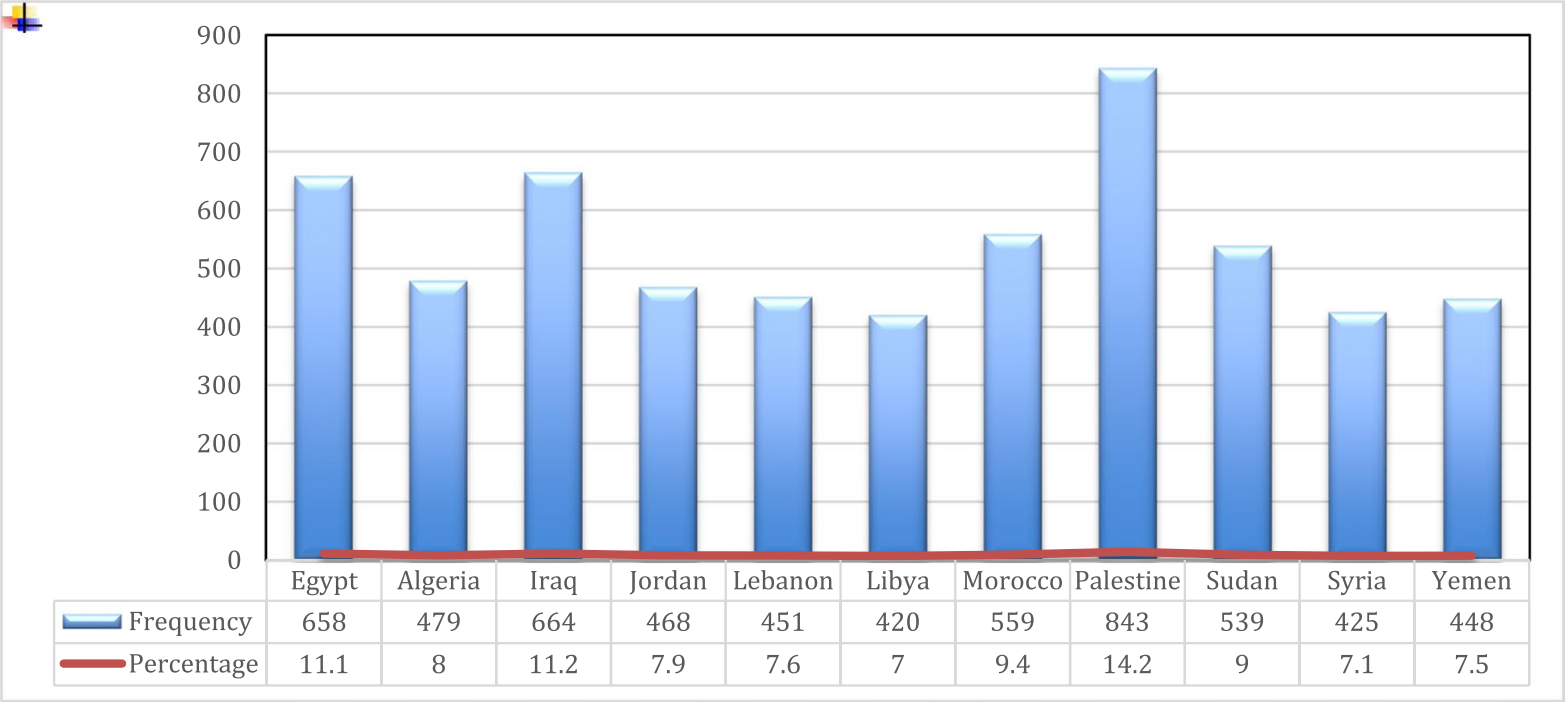
Flowchart of the study sample distribution across the Middle East and North Africa countries (*n*=5594)

In total, 8277 university students filled out the questionnaire. We excluded data from 2323 participants owing to incompatibility with our eligibility criteria. Of them, 3.7% (*n*=87) didn't provide informed consent, and 81.4% (*n*=1892) were suspected of having secondary headaches, based on a self-reported screening question at the beginning of the questionnaire. **(**Table S5**)** A total of 5,954 participants were included in the final analysis from 11 countries (Fig. 1).

Female participants represent 70.6%. Most participants (55.8%) were aged between 18 and 20 years and 68% were studying medicine. Among participants, 33.8% achieved a “Very Good"grade in the last academic year (Table 1)**. **

**Table 1:** Bivariable and multivariable modified poisson regression analysis of sociodemographic factors associated with migraine

**Variables**	**Total**		**Migraine Screening**				**Crude PR (95% CI)**	**Adjusted PR (95% CI)**
	(***n***=5954)		**Negative **(***n***=4398)		**Positive **(***n***=1556)			
	**n**	**%**	**n**	**%**	**n**	**%**		
Sex								
Female	4203	70.6	2879	68.5	1324	31.5	1	1
Male	1751	29.4	1519	86.8	232	13.2	0.42 (0.37, 0.48)**	0.41 (0.36, 0.47)**
Age (Years)								
18-20	3322	55.8	2486	74.8	836	25.2	1	1
21-23	1963	33.0	1454	74.1	509	25.9	1.03 (0.94, 1.13)	1.07 (0.98, 1.18)
24-30	669	11.2	458	68.5	211	31.5	1.25 (1.10, 1.42)**	1.3 (1.13, 1.50)**
Marital Status								
Currently married	359	6.0	253	70.5	106	29.5	1	1
Currently not married	5595	94.0	4145	74.1	1450	25.9	0.88 (0.74, 1.04)	1.2 (1.00, 1.45)
Family Income								
Not enough	1713	28.8	1256	73.3	457	26.7	1	1
Just enough	2434	40.9	1782	73.2	652	26.8	1 (0.91, 1.11)	0.98 (0.89, 1.09)
More than enough	1807	30.3	1360	75.3	447	24.7	0.93 (0.83, 1.04)	0.89 (0.80, 1.00)*
Field of Study								
Medical	4048	68.0	3022	74.7	1026	25.3	1	1
Non-Medical	1906	32.0	1376	72.2	530	27.8	1.1 (1.00, 1.20)*	1.14 (1.04, 1.25)**
The last academic degree								
Excellent	1727	29.0	1319	76.4	408	23.6	1	1
Very good	2012	33.8	1487	73.9	525	26.1	1.1 (0.99, 1.24)	1.05 (0.94, 1.17)
Good	1600	26.9	1164	72.8	436	27.2	1.15 (1.03, 1.30)*	1.05 (0.93, 1.18)
Fair	548	9.2	384	70.1	164	29.9	1.27 (1.09, 1.48)**	1.16 (0.99, 1.35)
Failed	67	1.1	44	65.7	23	34.3	1.45 (1.03, 2.05)*	1.38 (1.00, 1.91)

*PR *Prevalence ratio, *CI *Confidence interval

^*^P value significance ≤ 0.05

**P value <0.01

In the multivariate analysis, females had a significantly higher prevalence of suspected migraine compared to males. Compared to students aged 18–20 years, those aged 24–30 years had a significantly higher prevalence of suspected migraine. Participants who reported having “more than enough” income had a lower prevalence of migraine compared to those with “not enough” income. Students in non-medical fields had an increased prevalence of suspected migraine compared to medical students. No significant associations were found between the risk of migraine in relation to marital status and the last academic degree (Table 1).

Lifestyle characteristics revealed that 38.3% studied 1–3 hours daily and 34.6% reported no physical activity. More than half of the participants (55.8%) consumed 1–2 liters of fluids per day, 58.5% slept 7–8 hours per day, and 53.8% consumed caffeine-containing drinks daily. A family history of migraine was reported by 25.9%, and 9.3% had chronic diseases (Table 2).

**Table 2:** Bivariable and multivariable modified poisson regression analysis of lifestyle factors associated with migraine

Variables	Total		Migraine Screening				Crude PR (95% CI)	Adjusted PR (95% CI)
	(*n*=5954)		Negative (*n*=4398)		Positive (*n*=1556)			
	n	%	n	%	n	%		
Study hours								
less than one hour	678	11.4	466	68.7	212	31.3	1	1
1-3 hours/day	2283	38.3	1728	75.7	555	24.3	0.78 (0.68, 0.89)**	0.8 (0.70, 0.91)**
4-6 hours/day	2197	36.9	1640	74.6	557	25.4	0.81 (0.71, 0.93)**	0.83 (0.73, 0.94)**
7-9 hours/day	629	10.6	451	71.7	178	28.3	0.91 (0.77, 1.07)	0.89 (0.75, 1.04)
10 hours or more	167	2.8	113	67.7	54	32.3	1.03 (0.81, 1.32)	0.91 (0.72, 1.15)
Physical Activity								
No physical activity	2058	34.6	1436	69.8	622	30.2	1	1
Less than 1.5 h/w	1525	25.6	1136	74.5	389	25.5	0.84 (0.76, 0.94)**	0.88 (0.79, 0.98)*
1.5-3 h/w	1170	19.7	889	76	281	24	0.79 (0.70, 0.90)**	0.83 (0.74, 0.94)**
3-5 h/w	645	10.8	499	77.4	146	22.6	0.75 (0.64, 0.88)**	0.81 (0.69, 0.95)**
More than 5 h/w	556	9.3	438	78.8	118	21.2	0.7 (0.59, 0.83)**	0.77 (0.65, 0.91)**
Fluid Intake								
Less than 1 liter/day	1566	26.3	1036	66.2	530	33.8	1	1
1-2 liter/day	3324	55.8	2537	76.3	787	23.7	0.7 (0.64, 0.77)**	0.77 (0.70, 0.84)**
3-4 liter/day	911	15.3	712	78.2	199	21.8	0.65 (0.56, 0.74)**	0.71 (0.62, 0.82)**
More than 4 liter/day	153	2.6	113	73.9	40	26.1	0.77 (0.59, 1.02)	0.95 (0.72, 1.25)
Sleeping hours								
6 hours per day or less	1569	26.4	1064	67.8	505	32.2	1	1
7-8 h/day	3485	58.5	2670	76.6	815	23.4	0.73 (0.66, 0.80)**	0.76 (0.69, 0.83)**
More than 8 h/day	900	15.1	664	73.8	236	26.2	0.81 (0.71, 0.93)**	0.8 (0.71, 0.91)**
Smoking								
No	5383	90.4	3977	73.9	1406	26.1	1	
Yes	571	9.6	421	73.7	150	26.3	1.01 (0.87, 1.16)	0.95 (0.82, 1.09)
Daily Caffeine Consumption								
No	2751	46.2	2108	76.6	643	23.4	1	1
Yes	3203	53.8	2290	71.5	913	28.5	1.22 (1.12, 1.33)**	1.19 (1.09, 1.30)**
Chronic Diseases								
No	5403	90.7	4072	75.4	1331	24.6	1	1
Yes	551	9.3	326	59.2	225	40.8	1.66 (1.48, 1.85)**	1.46 (1.31, 1.62)**
Family History of Migraine								
No	4409	74.1	3484	79	925	21	1	1
Yes	1545	25.9	914	59.2	631	40.8	1.95 (1.79, 2.12)**	1.82 (1.67, 1.98)**

*PR *Prevalence ratio, *CI* Confidence interval

^*^P value significance ≤ 0.05

**P value <0.01

The multivariate analysis showed that those studying 1–3 hours on regular study days and those studying 4–6 hours had a significantly lower prevalence of suspected migraine compared to those who studied less than 1 hour. Those with regular physical activity are significantly less likely to experience from suspected migraine compared to those with no physical activity. Increased fluid intake and sleeping more than six hours were correlated with a statistically significant lower risk of migraine compared to students sleeping 6 hours or less per day. In contrast, daily caffeine- containing drink consumption was significantly associated with increased migraine risk. Students with chronic diseases and those with a positive family history of migraine had significantly higher migraine risk. Smoking had no significant association (Table 2).

**Fig. 2:**
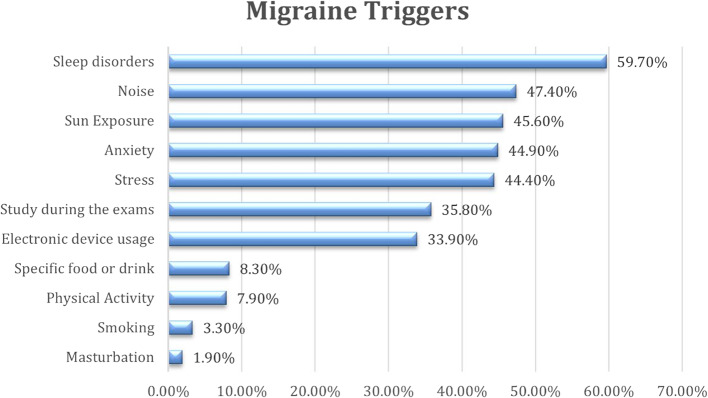
The distribution of self-reported migraine triggers among participants screened positive for migraine (*n* = 1556)

**Fig. 3:**
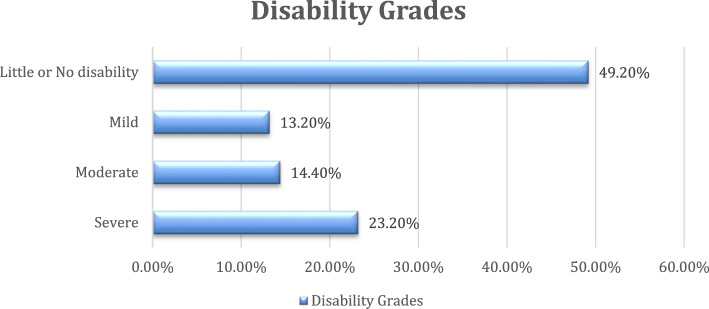
The migraine-related disability grades among participants screened positive for migraine (n = 1556).

Using the MS-Q, a total of 5,954 participants were screened for migraine. Of them, 26.1% (*n*=1556) had a total score of ≥ 4 indicating a likely diagnosis of migraine. Migraine screening showed varying prevalence across countries. The prevalence was found to be much higher in Iraq (38.9%), Libya (34.8%), and Algeria (31.5%). The crude prevalence ratios significantly increased: 1.95, 1.75, and 1.58, respectively (*p* < 0.01). Palestine (25.1%) and Syria (25.9%) had a higher migraine prevalence than Egypt. Other countries didn't demonstrate differences with statistical significance, including of Jordan, Lebanon, Sudan, and Yemen. Morocco had a slightly lower prevalence at 18.4% with no significant association (Table 3). Among participants who screened positive for migraine, sleeping disorders were the most frequently reported migraine trigger (59.7%) (Fig. 2). Disability assessment showed that 23.2% of participants had severe disability (Fig. 3).

**Table 3:** Migraine screening by country with corresponding prevalence ratios

**Country**	**Migraine Screening**				
	**Negative **(***n***=4398)		**Positive **(***n***=1556)		**Crude PR (95% CI)**
	**n**	**%**	**n**	**%**	
Egypt	527	80.1	131	19.9	1
Algeria	328	68.5	151	31.5	1.58 (1.29, 1.94)**
Iraq	406	61.1	258	38.9	1.95 (1.63, 2.34)**
Jordan	358	76.5	110	23.5	1.18 (0.94, 1.48)
Lebanon	352	78.0	99	22.0	1.1 (0.87, 1.39)
Libya	274	65.2	146	34.8	1.75 (1.43, 2.14)**
Morocco	456	81.6	103	18.4	0.93 (0.73, 1.17)
Palestine	631	74.9	212	25.1	1.26 (1.04, 1.53)*
Sudan	413	76.6	126	23.4	1.17 (0.95, 1.46)
Syria	315	74.1	110	25.9	1.3 (1.04, 1.62)*
Yemen	338	75.4	110	24.6	1.23 (0.99, 1.54)

*PR *Prevalence ratio, *CI *Confidence interval

^*^P value significance ≤ 0.05

**P value <0.01

Among positive cases, mental health comorbidities were notable 22.4% had moderately severe depression, 72.5% had severe depression, 29.3% had moderate anxiety, and 24.9% had severe anxiety (Table 4).

**Table 4:** Modified poisson regression analysis of migraine and its association with depression and anxiety

**Variables**	**Total**		**Migraine Screening**				**Crude PR (95% CI)**	**Adjusted PR (95% CI)**
	(***n***=5954)		**Negative **(***n***=4398)		**Positive# **(***n***=1556)			
	**n**	**%**	**n**	**%**	**n**	**%**		
Anxiety								
Minimal anxiety	1885	31.7	1667	88.4	218	11.6	1	1
Mild	1950	32.8	1464	75.1	486	24.9	2.16 (1.86, 2.50)**	2.2 (0.36, 13.4)
Moderate	1284	21.6	819	63.8	465	36.2	3.13 (2.71, 3.62)**	2.16 (0.37, 12.7)
Severe	835	14.0	448	53.7	387	46.3	4.01 (3.47, 4.63)**	2.57 (0.44, 15.1)
Depression								
Mild and Moderate	174	3.0	94	54.0	80	46.0	1	1
Moderately severe	804	13.5	456	56.7	348	43.3	0.94 (0.79, 1.13)	1.62 (0.26, 9.95)
Severe	4976	83.6	3848	77.3	1128	22.7	0.49 (0.42, 0.58)**	0.56 (0.10, 3.23)

*PR *Prevalence ratio, *CI *Confidence interval

^*^P value significance ≤ 0.05

**P value <0.01

^#^Notably, we calculated the percentage of depression and anxiety levels among participants who screened positive for migraine (*n* = 1,556). Among these, 14% had minimal anxiety, 31.2% mild, 29.9% moderate, and 24.9% severe anxiety. Levels of depression as follows, 0.1% mild depression, 5% moderate, 22.4% moderately severe, and 72.5% had severe depression.

Different levels of anxiety severity significantly increased the crude PR of migraine as compared to minimal anxiety. The adjusted crude PR was 2.16 for mild anxiety, 3.13 for moderate anxiety, and increased to 4.01 for severe anxiety (95% CI: 1.86-2.50, 2.71-3.62, and 3.47-4.63, respectively). Compared with mild and moderate depression, no significant associations were found across different depression levels (Table 4).

The interaction between anxiety and depression levels among migraine-positive students showed no significant results (Table S6). Moreover, a weak negative correlation between migraine disability score and academic achievements was observed (−0.069, *p* < 0.001) (Figure S1).

The prevalence of migraine was higher in upper-middle income countries, particularly among females, those with low physical activity, low water intake, poor sleep hours, high caffeine intake, and a family history of migraine. Medical students dominate among all countries with better academic grades in low and lower-middle-income countries. Psychological distress shows varied levels among countries. However, severe depression levels were predominant in all countries (Table S7).

## Discussion

### Prevalence of migraine

To our knowledge, this is the first study to comprehensively evaluate migraine prevalence and associated factors among university students in the MENA region. Previous research was conducted in individual MENA countries, such as Egypt, Oman, Kuwait, Saudi Arabia, Iraq, Iran, Turkey, and Syria, as summarized in a recent meta-analysis [18]. In contrast, our study encompasses a large, diverse sample, primarily from low- and middle-income MENA countries, and provides a broader perspective on this debilitating condition. Additionally, our study assessed key comorbidities such as depression and anxiety, along with different sociodemographic and lifestyle factors associated with migraine.

Our study identified a migraine prevalence of 26.1% among university students in low- and middle-income MENA countries. A recent meta-analysis reported a global migraine prevalence of 19% [95% CI: 16.1–21.8%] among university students worldwide [18]. The prevalence observed in our study exceeded that reported in Europe (18.8%, 95% CI: 12.8–25.8%), Africa (15.2%, 95% CI: 9.9–22.7%), Asia (16.3%, 95% CI: 13.1–20.2%), USA (22.5%, 95% CI: 12.4–37.3%), and Australia (25.7%,95% CI: 20.5–31.8%). However, it remained lower than the prevalence recorded in Latin America (27.9% [95% CI: 17.7–41.2%]) [18]. Taken together, the prevalence of migraine in the MENA region ranks as the second highest globally, indicating a substantial burden of the studied condition in this region. However, this should be approached cautiously because migraine prevalence differs greatly based on the diagnostic criteria applied. Furthermore, regional factors that may influence migraine development and progression such as environmental, and lifestyle triggers, dietary habits, genetic predisposition, cultural differences, socioeconomic stressors, psychological factors, metabolic factors, hormonal imbalances, underdiagnosed headache, low awareness, and healthcare access [11, 26, 27]. Additionally, consider the frequent obstacles that people with headache problems in LMICs encounter while trying to get a correct diagnosis and treatment for their headache such as political/economic barriers, inadequate treatment or therapeutic mismanagement, community misperceptions, and ineffective healthcare delivery systems [28].

In our study, we used the MS-Q tool to screen migraine which was developed based on the criteria of the International Headache Society (IHS). The MS-Q cutoff (≥4) demonstrated a sensitivity of 0.93 and specificity of 0.81, with Cronbach's alpha coefficient = 0.82 [29]. However, the previous meta-analysis primarily relied on the International Classification of Headache Disorders (ICHD) criteria and, to a lesser extent, the ID Migraine (IDM) tool in some studies [18]. Large-scale validations against ICHD criteria reported a sensitivity of 0.82 and specificity of 0.97, demonstrating the MS-Q’s accurate migraine detection with low false positive rates [30, 31]. In the MENA context, previous validation of MS-Q in primary care settings applied the ROC curve for MS-Q and ICHD-3 scores and showed an area under the curve (AUC) of 0.97 (95% CI, 0.94-0.99), with a sensitivity of 0.95, and specificity of 0.99 [20]. Furthermore, the Cronbach’s alpha in our study showed a value of 0.72 indicating acceptable internal consistency. The slightly lower values in our sample may be partly attributed to the limited number of items (5 items), as shorter scales tend to yield lower alpha values even when internal consistency is adequate [32]. However, this may modestly affect the accuracy of prevalence estimates. It could lead to a slight overestimation by identifying individuals with symptoms that do not fully meet diagnostic criteria, or a slight underestimation if some cases are missed due to limited inter-item correlation. Nevertheless, as this value exceeds the acceptable threshold, any resulting bias is likely minimal, and the MS-Q remains a valuable tool for migraine screening in population-based research. Besides these factors, other elements may help explain our results.

An intriguing finding is that Iraq had the highest migraine prevalence, followed by Libya, whereas Egypt and Morocco reported the lowest rates. Similarly, a meta-analysis and a narrative review suggested that the prevalence of migraine is higher in Asia than in African regions [18, 33]. These variations are consistent with previous findings that show migraine prevalence in the Middle East varies widely, ranging from 2.6% to 32%, and is affected by factors like dietary habits, sleep disorders, stress, and psychological comorbidities like depression and anxiety, which were common in the MENA region [11]. This disparity might be explained by differences in socioeconomic status, healthcare services, cultural perspective toward headache reporting, and psychological problems, in addition to the variations in the methodologies employed and the different migraine screening tools used.

Furthermore, many of these countries in our study have been in long-term war, political instability, and other conflicts like Iraq, Syria, and Libya. These stressors may contribute to elevated migraine prevalence and associated triggers, in addition to affects on healthcare systems and increased socio-economic hardships. On top of that, university students in MENA countries are facing political instability, economics, and academic workload, and often inadequate support systems, which may contribute to increased migraine triggers and prevalence. Furthermore, low- and middle-income status limits access to timely diagnosis and effective management, leading to underreported, underdiagnosed, and undertreated migraine [11, 28]. However, due to the limited availability of relevant literature, we recommend that future research explore these hypotheses to better understand the underlying causes of migraine prevalence variations among students in MENA countries to address migraine burden effectively in diverse MENA settings.

### Sociodemographic characteristics and their association with migraine

Among our participants, migraine was more prevalent in females, consistent with trends observed in the general population [34]. A recent meta-analysis revealed that the prevalence rate of migraines in females was double that of males among university students worldwide [18]. This sex and gender difference is attributed to hormonal effects, pain perception, brain structural and functional differences, genetic factors, and behavioral and dietary habits [35]. Furthermore, although a recent review found that migraine prevalence increases with age, peaking between 30 and 34 years in the general population [36], our findings suggested an earlier peak among university students, specifically between 24 and 30 years. Certain studies similarly reported that students over 20 years old experienced migraine significantly more frequently than their younger counterparts [5, 37]. This discrepancy may be attributed to unique stressors and lifestyle factors associated with students’ lives. Additionally, students with chronic diseases and a positive family history of migraine showed higher odds of developing migraines. These findings align with similar studies in both university students and the general population [5, 37–41]. A family history of migraine increases the risk due to genetic factors, with heritability estimated between 35% and 60% [42]. Furthermore, there is a bidirectional relationship between chronic diseases and migraines. While some chronic conditions increase the risk of migraine, migraine can also raise the risk of certain comorbidities, like depression, hypertension, and epilepsy. [43].

Interestingly, we observed that non-medical students had a significantly higher prevalence of migraine compared to medical students. This finding is consistent with a recent meta-analysis, which reported that students from mixed subject majors experienced migraines more frequently than those studying medicine [18]. One possible explanation is that medical students, despite their demanding coursework, may have better awareness of migraine triggers, healthier coping strategies, and a more structured routine, which could mitigate migraine occurrence.

### Associations between lifestyle factors and migraine

Regarding lifestyle characteristics, we found that students with higher fluid intake, more than 7 hours of sleep, and regular physical activity had a significantly lower migraine incidence. However, daily caffeine consumption was linked to a higher risk. Hydration is a cost-effective, non-invasive, and low-risk strategy to alleviate or avert headache pain [44]. Studies indicated that higher daily water intake is associated with decreased migraine severity, frequency, and duration [44–47]. Persistent mild dehydration may induce headaches while maintaining proper hydration serves as a key preventive strategy [44, 48]. The association between migraine and sleep disorders is complex and bidirectional with evidence supporting reverse causality [49]. While poor sleep is widely recognized as a trigger for migraine attacks, migraines themselves can disrupt normal sleep patterns through pain and neurological disturbances [50, 51]. Pansod et al indicated that migraine patients have significantly poorer sleep quality compared to controls and that sleep quality worsens as migraine severity increases, suggesting that migraine attacks directly impair sleep. Nonetheless, anecdotal evidence suggests that sleep may have a therapeutic impact on migraine relief [52]. Similarly, multiple studies have found that higher fluid intake, adequate sleep duration, and regular physical activity are associated with reduced migraine severity, duration, and frequency [13, 46, 47, 53, 54]. Regular exercise may alleviate migraine symptoms by enhancing stress resilience and increasing endorphin release, which plays a role in pain modulation [38, 41, 55]. Conversely, caffeine intake is more likely to worsen these migraine parameters by influencing neurotransmitter release and activating specific neuronal pathways [54].

Many students develop headaches while studying. While there is no direct link between migraine and studying, many of the activity’s students engage in while studying can cause migraine, like lacking sleep, eating unhealthy snacks, and consuming lots of coffee. Our study demonstrated that 1-6 hours of studying reduced the risk of migraine, possibly due to balanced cognitive activity. Consistent with our findings, research indicates that individuals with higher grade point averages tend to have a significantly lower prevalence of migraine [56]. Students with lower grades may experience higher levels of stress and workload, potentially leading to increased migraine triggers. Lifestyle refers to a person’s-controlled behaviors and activities, which can entail risk factors. Thus, changing these lifestyle factors is important to mitigate the frequency and severity of migraines.

### Migraine triggers

Results of the present study revealed that the primary migraine triggers among MENA university students included sleep disturbances, noise, and sun exposure. In a study among Bangladeshi university students, stress and irregular sleep were the main triggers of migraine attacks, followed by academic pressure, external noise, and sun exposure [39]. Likewise, sleep disturbances were noted to be the major trigger of migraines among university students worldwide [39, 40, 57]. These findings align with the results of studies conducted among university students of Kuwait [58], the University of Gondar in Ethiopia [59], university students in the United States [60], and Brazil [61]. To effectively manage migraines, it is essential to comprehend these triggers.

### Psychological comorbidities with migraine

Multiple studies indicated that anxiety and depression are the most prevalent psychiatric comorbidities in migraine cases, even after adjusting for age, gender, and other confounding factors [15, 39, 62, 63]. Consistent with this, our results revealed a high prevalence of anxiety and depression. Similarly, a systematic review reported elevated rates of anxiety and depression in university populations worldwide, often exceeding those in the general population [64]. For example, a study in Bangladesh reported that students experiencing anxiety faced a 2.35-fold increased risk of developing migraine, while those with depressive symptoms had a 1.35-fold higher risk [39]. The imperative implications of these findings necessitate institutional responses for integrated mental health support in MENA university settings, including early screenings and mental health promotion activities, due to the high burden of anxiety and depression and their link to migraine among students.

Furthermore, several studies agree that the severity of anxiety can influence migraine intensity, while they have disagreed regarding the impact of depression [15, 62, 65–68]. Although Chu et al. [68] and Bintari et al. [66] found a significant correlation between depression levels and migraine severity. Lee et al. did not report similar results [65], which is consistent with our findings. Anxiety and depression are thought to have increased among healthy university students over the past few decades, potentially contributing to the rising prevalence of migraine [69, 70]. This trend suggests a complex interaction between migraine, depression, anxiety, and university student experience where individuals with migraine are more likely to develop anxiety and depression over time, and vice versa [71].

Despite previous literature supporting the complex relationship between depression, anxiety, and migraine, our study showed no significant associations after controlling confounding. This discrepancy may be due to the presence of stigma and low mental health literacy in MENA populations, the symptoms may be misreported or misclassified, creating problems for measurement with the PHQ-9 and GAD-7 and a formal statistical association with migraine risk [72, 73]. However, our findings should be approached cautiously due to the cross-sectional design nature. Thus, additional longitudinal research is needed to better understand the complex relationships observed.

### Migraine related disability

According to our results, 23.2% of participants suffered from severe disability due to migraine. In a study in South America, 75.7% of university students with suspected migraine experienced functional disability, with the majority exhibiting severe levels [74]. Similarly, in Saudi Arabia, a study found that over half of the participants experienced severe disability due to migraine, while 20.7% reported moderate disability [75]. These findings have also been confirmed in several other studies involving university students [76, 77]. Migraine headaches affect quality of life by leading to missed university or work days, decreased productivity, and creating financial strain due to the cost of medications [78].

Furthermore, we found a weak negative correlation between migraine disability scores and academic achievement. The negative correlation that exists between academic achievement and migraine disability is weak statistically but may still be of practical relevance, especially in a highly competitive academic environment. Epidemiological data revealed a 62.7% reduction in performance among university students with migraine [79]. Similarly, a recent review shows that migraine attacks negatively affect educational performance, with students reporting reduced functioning or college absenteeism ranging from one to 20 days. However, it is worth noting that many students continue to attend classes despite suffering from migraines [80]. This demonstrates their dedication or a potential lack of flexibility in the academic program. Our findings are consistent with disability related to migraine affecting concentration, attendance, and overall performance, and highlight the necessity to create university plans to help migraine sufferers continue to perform well academically.

The literature states that migraine incidence is higher among females, middle-aged people, and people with low income and socioeconomic status. Moreover, there was evidence that migraine is more prevalent in persons with lower incomes but vary as much to remission as that of higher income people [81, 82]. Perhaps that is because low socioeconomic status is associated with increased stressful life events, poor access to health care, and other adverse social determinants [81]. Dissatisfaction with one's income regardless of income level makes one more prone to migraine, but the reasons for such dissatisfaction may vary [83]. Similarly, our study showed that participants who reported having"more than enough"income had a lower likelihood of screening positive for migraine compared to those with"not enough"income. Meanwhile, our study included low and middle-income countries; a comparison of sociodemographic and lifestyle characteristics among these countries grouped by socioeconomic status according to the World Bank showed that females were consistently more affected in all groups, supporting previous literature. Differences in lifestyle factors and mental health indicators highlight the multifactorial character of migraine and socioeconomic, biological, and environmental context interaction. The findings thus support the development of area- and economy-specific interventions to effectively control migraine in the MENA region.

### Future implications and recommendations

In line with previous studies, university students in the MENA region seem to experience a significant prevalence of migraines, with depression and anxiety negatively impacting their academic achievement. Thus, awareness should be raised regarding the mental health challenges students face and how these can impact physical health to provide better support and interventions. Alongside standard migraine evaluations, mental health screens should be implemented to effectively identify and manage comorbid problems. Based on the observed associations between migraine and factors such as good hydration, regular physical activity, and adequate good sleep hours which could decrease migraine risk, programs that enhance awareness about the importance of these lifestyle modifications must be adopted. Additionally, our findings demonstrated that stress and anxiety are among the most common migraine triggers, therefore stress management programs should be developed to teach students effective stress-relief techniques, which can also contribute to better sleep. More attention should be given to alleviating students'academic pressures and reducing their working hours in a simple and manageable way. Furthermore, a consensus for annual screening of university students should be implemented to identify migraines, particularly for students with low grades. However, we recommend that depression and anxiety should not be ignored when evaluating migraine patients to ensure good prognosis and effective management of the condition. Additionally, during a migraine attack, students should be provided with appropriate advice on managing their headaches, how to avoid migraine triggers, accessing medical care effectively, and preventing drug misuse, overuse, and other complications. Future longitudinal research to assess migraine prevalence using more rigorous and representative sampling methods, to explore sociodemographic and lifestyle factors influencing migraine, and to investigate the causal links between migraines and their comorbidities are recommended.

### Strengths and limitations

We explored a variety of factors, including sociodemographic, lifestyle, and psychological aspects, to better understand migraine's varied effects on health. We anticipate that the findings will contribute to a better understanding of the epidemiology of migraines among university students in this region. Additionally, it may guide the development of targeted interventions and preventative strategies to improve the management and outcomes of migraine sufferers in the MENA region. However, certain limitations of the study should be acknowledged. Firstly, given the cross-sectional design of the study, causality cannot be inferred from the observed associations. The direction and timing of these relationships remain uncertain. Longitudinal research is needed to clarify temporal sequences and explore potential causal links. Furthermore, the study used non-probability sampling methods (convenience and snowball sampling). While these methods enable us to access a wide and diverse group of university students from 11 countries, we acknowledge that the participants may not fully represent the broader student group in participating countries due to potential selection biases. Although we aimed for balanced recruitment, differences in sample size among countries may have introduced regional bias, limiting generalizability to the entire MENA student population. Although the MS-Q and MIDAS questionnaires used in this study were previously validated with high internal consistency, the Cronbach alpha value observed in our pilot study was acceptable but slightly low. The use of self-administered questionnaires introduces potential risks of recall bias, response bias, and non-response bias, which may lead to either under-reporting or over-reporting of prevalence by participants. To mitigate these biases, the questionnaire was kept anonymous, clearly worded, and pilot-tested to ensure clarity and comprehension. Since MS-Q is a screening test, it might not illuminate all the complexities around the migraine diagnostic process; hence future studies using probability sampling methods, clinical evaluations, and longitudinal designs are recommended to validate and extend these findings and provide a clearer picture of migraine prevalence and its associated mental health conditions across different MENA countries.

## Conclusion

Migraine was highly prevalent among university students in low- and middle-income MENA countries, significantly affecting students' daily activities, mental health, and academic performance. Protective factors against migraines include higher fluid intake, sufficient sleep, extended study hours, and regular physical activity, while daily caffeine-containing drinks consumption increases the risk. Sleep disturbances, noise, sun exposure, and stress were identified as the most common triggers. Anxiety and depression were frequently observed in students with migraines, with anxiety being a key factor in migraine severity. These findings highlight the need for targeted interventions including health education programs for university students about migraine symptoms, its triggers, and comorbidities, as lifestyle and behavior modification programs to improve the academic success and overall well-being of university students in the MENA.

## Supplementary Information

Supplementary Material 1

## Data Availability

Data is available at the reasonable request of the corresponding author.

## Abbreviations

GBD: The Global Burden of Disease
YLDs: Years Lived with Disability
MENA: Middle East and North Africa.
MS-Q: Migraine Screening Questionnaire.
STROBE: The Strengthening of the Reporting of Observational Studies in Epidemiology.
CDC: Center for Disease Control and Prevention.
MIDAS: Migraine Disability Assessment Score.
GAD-7: Generalized Anxiety Disorder 7-item.
PHQ-9: Patient Health Questionnaire.
PRs: Prevalence ratios
CI: Confidence intervals
ICHD: International Classification of Headache Disorders
