# Supplementary material for: Burden of migraine among university students in the Middle East and North Africa: a cross-sectional study of prevalence, mental health comorbidities, and disability
Source: BMC Public Health. 2025 Oct 27;25:3605. doi: 10.1186/s12889-025-24293-9 (PMC12557868; doi:10.1186/s12889-025-24293-9)
Supplement: Supplementary file 1 — Supplementary Material 1 [file 12889_2025_24293_MOESM1_ESM.docx]

**Supplemental Appendix**

**Burden of Migraine Among University Students in Middle East and North Africa: A Cross-Sectional Study of Prevalence, Mental Health Comorbidities, and Disability**

**Supplemental Appendix content**

| **Table S1: Distribution of participants by country and migraine screening results with corresponding odds ratios** |
| --- |
| **Table S2: Lifestyle characteristics of study participants and their associations with migraine** |
| **Table S3: Migraine and its association with depression and anxiety** |
| **Table S4: Distribution of participants by country and migraine screening results with corresponding odds ratios** |
| **Table S5: Frequency and percentage of secondary headache causes** |
| **Table S6: The interaction between anxiety and depression levels, and migraine** |
| **Table S7:** **Comparison of Sociodemographic and Lifestyle Characteristics Among MENA Countries Grouped by Socioeconomic Status** |
| **Figure S1: Correlation between migraine disability score and academic achievements** |

**Table S1: Sociodemographic characteristics of study participants and their associations with migraine**

| **Variables** | **Total (n=5954)** | | **Migraine Screening** | | | | **OR (95% CI, P-value) (univariable)** | **aOR (95% CI, P-value) (multivariable)** |
| --- | --- | --- | --- | --- | --- | --- | --- | --- |
|  |  |  | **Negative**  **(n=4398)** | | **Positive**  **(n=1556)** | |  |  |
|  | **n** | **%** | **n** | **%** | **n** | **%** |  |  |
| **Sex** |  |  |  |  |  |  |  |  |
| Female | 4203 | 70.6 | 2879 | 68.5 | 1324 | 31.5 | 1 | 1 |
| Male | 1751 | 29.4 | 1519 | 86.8 | 232 | 13.2 | 0.33 (0.28-0.39)** | 0.32 (0.27-0.37)** |
| **Age** |  |  |  |  |  |  |  |  |
| 18-20 | 3322 | 55.8 | 2486 | 74.8 | 836 | 25.2 | 1 | 1 |
| 21-23 | 1963 | 33.0 | 1454 | 74.1 | 509 | 25.9 | 1.04 (0.92-1.18) | 1.11 (0.97-1.26) |
| 24-30 | 669 | 11.2 | 458 | 68.5 | 211 | 31.5 | 1.37 (1.14-1.64)** | 1.47 (1.19-1.80)** |
| **Marital Status** |  |  |  |  |  |  |  |  |
| Currently married | 359 | 6.0 | 253 | 70.5 | 106 | 29.5 | 1 | 1 |
| Currently not married | 5595 | 94.0 | 4145 | 74.1 | 1450 | 25.9 | 0.83 (0.66-1.06) | 1.30 (0.99-1.70) |
| **Family Income** |  |  |  |  |  |  |  |  |
| Not enough | 1713 | 28.8 | 1256 | 73.3 | 457 | 26.7 | 1 | 1 |
| Just enough | 2434 | 40.9 | 1782 | 73.2 | 652 | 26.8 | 1.01 (0.87-1.16) | 0.97 (0.84-1.13) |
| More than enough | 1807 | 30.3 | 1360 | 75.3 | 447 | 24.7 | 0.90 (0.78-1.05) | 0.85 (0.73-1.00)* |
| **Field of Study** |  |  |  |  |  |  |  |  |
| Medical | 4048 | 68.0 | 3022 | 74.7 | 1026 | 25.3 | 1 | 1 |
| Non-Medical | 1906 | 32.0 | 1376 | 72.2 | 530 | 27.8 | 1.13 (1.00-1.28)* | 1.21 (1.06-1.37)** |
| **The last academic degree** |  |  |  |  |  |  |  |  |
| Excellent | 1727 | 29.0 | 1319 | 76.4 | 408 | 23.6 | 1 | 1 |
| Very good | 2012 | 33.8 | 1487 | 73.9 | 525 | 26.1 | 1.14 (0.98-1.33) | 1.07 (0.92-1.25) |
| Good | 1600 | 26.9 | 1164 | 72.8 | 436 | 27.2 | 1.21 (1.04-1.42)* | 1.07 (0.91-1.26) |
| Fair | 548 | 9.2 | 384 | 70.1 | 164 | 29.9 | 1.38 (1.11-1.71)** | 1.23 (0.98-1.53) |
| Failed | 67 | 1.1 | 44 | 65.7 | 23 | 34.3 | 1.69 (0.99-2.80)* | 1.61 (0.93-2.71) |

*** P value significance ≤ 0.05.**P value <0.01. Abbreviations: OR (Odds ratio), aOR (adjusted odds ratio), CI (Confidence interval)**

**Table S2: Lifestyle characteristics of study participants and their associations with migraine**

| **Variables** | **Total (n=5954)** | | **Migraine Screening** | | | | **OR (95% CI, P-value) (univariable)** | **aOR (95% CI, P-value) (multivariable)** |
| --- | --- | --- | --- | --- | --- | --- | --- | --- |
|  |  |  | **Negative**  **(n=4398)** | | **Positive**  **(n=1556)** | |  |  |
|  | **n** | **%** | **n** | **%** | **n** | **%** |  |  |
| **Study hours** |  |  |  |  |  |  |  |  |
| less than one hour | 678 | 11.4 | 466 | 68.7 | 212 | 31.3 | 1 | 1 |
| 1-3 hours/day | 2283 | 38.3 | 1728 | 75.7 | 555 | 24.3 | 0.71 (0.59-0.85)** | 0.76 (0.62-0.93)** |
| 4-6 hours/day | 2197 | 36.9 | 1640 | 74.6 | 557 | 25.4 | 0.75 (0.62-0.90)** | 0.79 (0.64-0.97)* |
| 7-9 hours/day | 629 | 10.6 | 451 | 71.7 | 178 | 28.3 | 0.87 (0.68-1.10) | 0.89 (0.69-1.14) |
| 10 hours or more | 167 | 2.8 | 113 | 67.7 | 54 | 32.3 | 1.05 (0.73-1.50) | 0.84 (0.57-1.24) |
| **Physical Activity** |  |  |  |  |  |  |  |  |
| No physical activity | 2058 | 34.6 | 1436 | 69.8 | 622 | 30.2 | 1 | 1 |
| Less than 1.5 h/w | 1525 | 25.6 | 1136 | 74.5 | 389 | 25.5 | 0.79 (0.68-0.92)** | 0.89 (0.76-1.05) |
| 1.5-3 h/w | 1170 | 19.7 | 889 | 76.0 | 281 | 24.0 | 0.73 (0.62-0.86)** | 0.84 (0.70-1.00)* |
| 3-5 h/w | 645 | 10.8 | 499 | 77.4 | 146 | 22.6 | 0.68 (0.55-0.83)** | 0.79 (0.63-0.99)* |
| More than 5 h/w | 556 | 9.3 | 438 | 78.8 | 118 | 21.2 | 0.62 (0.50-0.78)** | 0.74 (0.58-0.94)* |
| **Fluid Intake** |  |  |  |  |  |  |  |  |
| Less than 1 liter/day | 1566 | 26.3 | 1036 | 66.2 | 530 | 33.8 | 1 | 1 |
| 1-2 liter/day | 3324 | 55.8 | 2537 | 76.3 | 787 | 23.7 | 0.61 (0.53-0.69)** | 0.73 (0.64-0.85)** |
| 3-4 liter/day | 911 | 15.3 | 712 | 78.2 | 199 | 21.8 | 0.55 (0.45-0.66)** | 0.68 (0.56-0.84)** |
| More than 4 liter/day | 153 | 2.6 | 113 | 73.9 | 40 | 26.1 | 0.69 (0.47-1.00) | 1.00 (0.66-1.49) |
| **Sleeping hours** |  |  |  |  |  |  |  |  |
| 6 hours per day or less | 1569 | 26.4 | 1064 | 67.8 | 505 | 32.2 | 1 | 1 |
| 7-8 h/day | 3485 | 58.5 | 2670 | 76.6 | 815 | 23.4 | 0.64 (0.56-0.73)** | 0.74 (0.64-0.85)** |
| More than 8 h/day | 900 | 15.1 | 664 | 73.8 | 236 | 26.2 | 0.75 (0.62-0.90)** | 0.76 (0.63-0.93)** |
| **Smoking** |  |  |  |  |  |  |  |  |
| No | 5383 | 90.4 | 3977 | 73.9 | 1406 | 26.1 | 1 |  |
| Yes | 571 | 9.6 | 421 | 73.7 | 150 | 26.3 | 1.01 (0.83-1.22) | Excluded# |
| **Daily Caffeine Consumption** |  |  |  |  |  |  |  |  |
| No | 2751 | 46.2 | 2108 | 76.6 | 643 | 23.4 | 1 | 1 |
| Yes | 3203 | 53.8 | 2290 | 71.5 | 913 | 28.5 | 1.31 (1.16-1.47)** | 1.22 (1.07-1.38)** |
| **Chronic Diseases** |  |  |  |  |  |  |  |  |
| No | 5403 | 90.7 | 4072 | 75.4 | 1331 | 24.6 | 1 | 1 |
| Yes | 551 | 9.3 | 326 | 59.2 | 225 | 40.8 | 2.11 (1.76-2.53)** | 1.62 (1.33-1.97)** |
| **Family History of Migraine** |  |  |  |  |  |  |  |  |
| No | 4409 | 74.1 | 3484 | 79.0 | 925 | 21.0 | 1 | 1 |
| Yes | 1545 | 25.9 | 914 | 59.2 | 631 | 40.8 | 2.60 (2.30-2.95)** | 2.44 (2.14-2.77)** |

*** P value significance ≤ 0.05.**P value <0.01, Excluded# for being insignificant in bivariate analysis. Abbreviations: OR (Odds ratio), aOR (adjusted odds ratio), CI (Confidence interval)**

**Table S3: Migraine and its association with depression and anxiety**

| **Variables** | **Total (n=5954)** | | **Migraine Screening** | | | | **OR (95% CI, P-value) (univariable)** | **aOR (95% CI, P-value) (multivariable)** |
| --- | --- | --- | --- | --- | --- | --- | --- | --- |
|  |  |  | **Negative**  **(n=4398)** | | **Positive**  **(n=1556)** | |  |  |
|  | **n** | **%** | **n** | **%** | **n** | **%** |  |  |
| **Anxiety** |  |  |  |  |  |  |  |  |
| Minimal anxiety | 1885 | 31.7 | 1667 | 88.4 | 218 | 11.6 | 1 | 1 |
| Mild | 1950 | 32.8 | 1464 | 75.1 | 486 | 24.9 | 2.54 (2.13-3.03)** | 2.23 (1.87-2.67)** |
| Moderate | 1284 | 21.6 | 819 | 63.8 | 465 | 36.2 | 4.34 (3.63-5.21)** | 3.65 (3.04-4.40)** |
| Severe | 835 | 14.0 | 448 | 53.7 | 387 | 46.3 | 6.61 (5.44-8.05)** | 5.15 (4.21-6.31)** |
| **Depression** |  |  |  |  |  |  |  |  |
| Mild | 10 | 0.2 | 8 | 80.0 | 2 | 20.0 | 1 |  |
| Moderate | 164 | 2.8 | 86 | 52.4 | 78 | 47.6 | 3.63 (0.88-24.52) | Excluded# |
| Moderately severe | 804 | 13.5 | 456 | 56.7 | 348 | 43.3 | 3.05 (0.76-20.31) | Excluded# |
| Severe | 4976 | 83.6 | 3848 | 77.3 | 1128 | 22.7 | 1.17 (0.29-7.78) | Excluded# |

*** P value significance ≤ 0.05.**P value <0.01, Excluded# for being insignificant in bivariate analysis. Abbreviations: OR (Odds ratio), aOR (adjusted odds ratio), CI (Confidence interval)**

**Table S4: Distribution of participants by country and migraine screening results with corresponding odds ratios**

| **Country** | **Total (n=5954)** | | **Migraine Screening** | | | | **OR (95% CI, P-value) (univariable)** |
| --- | --- | --- | --- | --- | --- | --- | --- |
|  |  |  | **Negative**  **(n=4398)** | | **Positive**  **(n=1556)** | |  |
|  | **n** | **%** | **n** | **%** | **n** | **%** |  |
| **Egypt** | 658 | 11.1 | 527 | 80.1 | 131 | 19.9 | 1 |
| **Algeria** | 479 | 8.0 | 328 | 68.5 | 151 | 31.5 | 1.85 (1.41-2.43)** |
| **Iraq** | 664 | 11.2 | 406 | 61.1 | 258 | 38.9 | 2.56 (2.00-3.28)** |
| **Jordan** | 468 | 7.9 | 358 | 76.5 | 110 | 23.5 | 1.24 (0.93-1.65) |
| **Lebanon** | 451 | 7.6 | 352 | 78.0 | 99 | 22.0 | 1.13 (0.84-1.52) |
| **Libya** | 420 | 7.0 | 274 | 65.2 | 146 | 34.8 | 2.14 (1.63-2.83)** |
| **Morocco** | 559 | 9.4 | 456 | 81.6 | 103 | 18.4 | 0.91 (0.68-1.21) |
| **Palestine** | 843 | 14.2 | 631 | 74.9 | 212 | 25.1 | 1.35 (1.06-1.73)* |
| **Sudan** | 539 | 9.0 | 413 | 76.6 | 126 | 23.4 | 1.23 (0.93-1.62) |
| **Syria** | 425 | 7.1 | 315 | 74.1 | 110 | 25.9 | 1.40 (1.05-1.88)* |
| **Yemen** | 448 | 7.5 | 338 | 75.4 | 110 | 24.6 | 1.31 (0.98-1.75) |

*** P value significance ≤ 0.05.**P value <0.01**

**Table S5: Frequency and percentage of secondary headaches causes**

| **Secondary headaches cause** | **N** | **%** |
| --- | --- | --- |
| **Chronic Sinusitis** | **1217** | **64.3** |
| **Hypertension** | **455** | **24.0** |
| **Head injury** | **153** | **8.1** |
| **Hydrocephalus** | **32** | **1.7** |
| **brain hemorrhage** | **22** | **1.2** |
| **Tumor in the brain** | **9** | **0.5** |
| **Operation in the head** | **4** | **0.2** |

**Table S6: The interaction between anxiety, depression levels, and migraine**

|  | | **Depression (Mild or Moderate as the reference level)** | |
| --- | --- | --- | --- |
|  |  | **Moderately severe Adjusted PR (95% CI)** | **Severe**  **Adjusted PR (95% CI)** |
| **Anxiety (Minimal anxiety as the reference level)** | **Mild** | 0.44 (0.07, 2.86) | 0.99 (0.16, 6.05) |
|  | **Moderate** | 0.62 (0.10, 3.86) | 1.39 (0.24, 8.26) |
|  | **Severe** | 0.6 (0.10, 3.75) | 1.49 (0.25, 8.80) |

**Table S7: Comparison of Sociodemographic and Lifestyle Characteristics Among MENA Countries Grouped by Socioeconomic Status**

| **Variables /countries** | **Low-income** | | **Lower-middle**  **income** | | **Upper-middle income** | | **P-value** |
| --- | --- | --- | --- | --- | --- | --- | --- |
| **Countries** | **Sudan, Syria, Yemen** | | **Egypt, Jordan, Lebanon, Morocco, Palestine** | | **Algeria, Iraq, Libya** | |  |
|  | **n** | **%** | **n** | **%** | **n** | **%** |  |
| **Total participants** | 1412 | 23.7 | 2979 | 50.0 | 1563 | 26.3 |  |
| **Positive migraine** | 346 | 24.5 | 655 | 22.0 | 555 | 35.5 | <0.001* |
| **Sex** |  |  |  |  |  |  | <0.001* |
| Female | 882 | 62.5 | 1999 | 67.1 | 1322 | 84.5 |  |
| Male | 530 | 37.5 | 980 | 32.9 | 241 | 15.5 |  |
| **Age** |  |  |  |  |  |  | <0.001* |
| 18-20 | 611 | 43.3 | 2032 | 68.2 | 679 | 43.5 |  |
| 21-23 | 627 | 44.4 | 762 | 25.6 | 574 | 36.7 |  |
| 24-30 | 174 | 12.3 | 185 | 6.2 | 310 | 19.8 |  |
| **Marital status** |  |  |  |  |  |  | <0.001* |
| Currently married | 72 | 5.1 | 128 | 4.3 | 159 | 10.2 |  |
| Currently not married | 1340 | 94.9 | 2851 | 95.7 | 1404 | 89.8 |  |
| **Income** |  |  |  |  |  |  | <0.001* |
| Not enough | 352 | 24.9 | 954 | 32.0 | 407 | 26.0 |  |
| Just enough | 662 | 46.9 | 1162 | 39.0 | 610 | 39.0 |  |
| More than enough | 398 | 28.2 | 863 | 29.0 | 546 | 35.0 |  |
| **Field of Study** |  |  |  |  |  |  | <0.001* |
| Medical | 1038 | 73.5 | 1856 | 62.3 | 1154 | 73.8 |  |
| Non-Medical | 374 | 26.5 | 1123 | 37.7 | 409 | 26.2 |  |
| **The last academic degree you obtained** | | |  |  |  |  | <0.001* |
| Excellent | 546 | 38.7 | 932 | 31.3 | 249 | 15.9 |  |
| Very good | 521 | 36.9 | 1046 | 35.1 | 445 | 28.5 |  |
| Good | 262 | 18.6 | 732 | 24.6 | 606 | 38.8 |  |
| Fair | 74 | 5.2 | 239 | 8.0 | 235 | 15.0 |  |
| Failed | 9 | 0.6 | 30 | 1.0 | 28 | 1.8 |  |
| **Studying (hours per day)** |  |  |  |  |  |  | <0.001* |
| less than one hour | 196 | 13.9 | 312 | 10.5 | 170 | 10.9 |  |
| 1-3 hours | 617 | 43.7 | 1069 | 35.9 | 597 | 38.2 |  |
| 4-6 hours | 468 | 33.1 | 1158 | 39.8 | 571 | 36.5 |  |
| 7-9 hours | 101 | 7.2 | 353 | 11.8 | 175 | 11.2 |  |
| 10 hours or more | 30 | 2.1 | 87 | 2.9 | 50 | 3.2 |  |
| **Physical Activity (hours per week)** | |  |  |  |  |  | <0.001* |
| No physical activity | 529 | 37.5 | 904 | 30.3 | 625 | 40.0 |  |
| Less than 1.5 h/w | 364 | 25.8 | 790 | 26.5 | 371 | 23.7 |  |
| 1.5-3 h/w | 260 | 18.4 | 637 | 21.4 | 273 | 17.5 |  |
| 3-5 h/w | 147 | 10.4 | 331 | 11.1 | 167 | 10.7 |  |
| More than 5 h/w | 112 | 7.9 | 317 | 10.6 | 127 | 8.1 |  |
| **Fluid Intake (liter per day)** | |  |  |  |  |  | <0.001* |
| Less than 1 liter/day | 287 | 20.3 | 786 | 26.4 | 493 | 31.5 |  |
| 1-2 liter/day | 834 | 59.1 | 1658 | 55.7 | 832 | 53.2 |  |
| 3-4 liter/day | 250 | 31.9 | 461 | 15.5 | 200 | 12.8 |  |
| More than 4 liter/day | 41 | 2.9 | 74 | 2.5 | 38 | 2.4 |  |
| **Sleeping (hours per day)** |  |  |  |  |  |  | <0.001* |
| 6 hours per day or less | 353 | 25.0 | 789 | 26.5 | 427 | 27.3 |  |
| 7-8 h/day | 876 | 62.0 | 1756 | 58.9 | 853 | 54.6 |  |
| More than 8 h/day | 183 | 13.0 | 434 | 14.7 | 283 | 18.1 |  |
| **Smoking** | 131 | 9.3 | 346 | 11.6 | 94 | 6.0 | <0.001* |
| **Daily Caffeine Consumption** | 745 | 52.8 | 1549 | 52.0 | 909 | 85.2 | <0.001* |
| **Chronic Diseases** | 122 | 8.6 | 242 | 8.1 | 187 | 12.0 | <0.001* |
| **Family History of migraine** | 349 | 24.7 | 662 | 22.2 | 534 | 34.2 | <0.001* |
| **Anxiety levels** |  |  |  |  |  |  | <0.001* |
| Minimal anxiety | 518 | 36.7 | 948 | 31.8 | 419 | 26.8 |  |
| Mild | 470 | 33.3 | 988 | 33.2 | 492 | 31.5 |  |
| Moderate | 263 | 18.6 | 641 | 21.5 | 380 | 24.3 |  |
| Severe | 161 | 11.4 | 402 | 13.5 | 272 | 17.4 |  |
| **Depression levels** |  |  |  |  |  |  | 0.014 |
| Mild | 1 | 0.1 | 9 | 0.3 | 0 | 0 |  |
| Moderate | 36 | 2.6 | 86 | 2.9 | 42 | 2.9 |  |
| Moderately severe | 180 | 12.7 | 378 | 12.7 | 246 | 15.7 |  |
| Severe | 1195 | 84.6 | 2506 | 84.1 | 1275 | 80.4 |  |

*** P value significance ≤ 0.05**


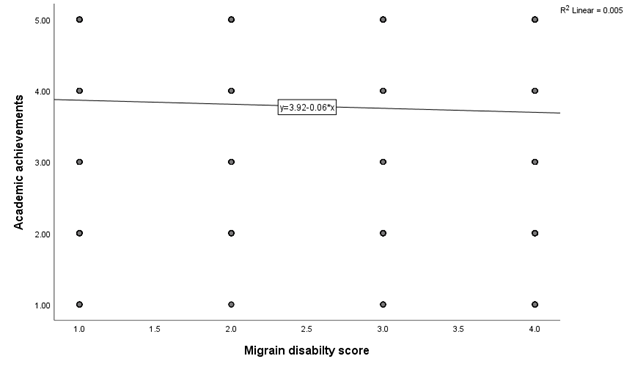


**Figure S1: Correlation between migraine disability score and academic achievements**
